# Labial Sensory Organs of Two *Leptoglossus* Species (Hemiptera: Coreidae): Their Morphology and Supposed Function

**DOI:** 10.3390/insects14010030

**Published:** 2022-12-28

**Authors:** Artur Taszakowski, Adrian Masłowski, Jolanta Brożek

**Affiliations:** Institute of Biology, Biotechnology and Environmental Protection, Faculty of Natural Sciences, University of Silesia in Katowice, Bankowa 9, 40-007 Katowice, Poland

**Keywords:** sensilla, Heteroptera, true bugs, leaf-footed bugs, *Leptoglossus occidentalis*, *Leptoglossus zonatus*

## Abstract

**Simple Summary:**

Several *Leptoglossus* Guérin-Méneville, 1831 species are important plant pests. *Leptoglossus occidentalis* (western conifer seed bug), a native of western North America, has become a highly successful worldwide invader that feeds on conifer cones and can be a significant pest in conifer seed orchards. *Leptoglossus zonatus,* or the large-legged bug, occurs throughout much of the Western Hemisphere and is an important pest to a wide range of crops in the southern United States. Mouthparts are necessary appendages specialised in detecting food sources and feeding. The types and quantities of various sensory organs are closely related to the feeding habits of insects. The labial sensilla occurring in the two aforementioned species were examined using field emission scanning electron microscopy, and their structural details were described for the first time. The elongated and slender, four-segmented labia have the same structure in both species. Nine types of aporous sensilla, whose function is probably mechanoreception, and three uniporous sensilla, most likely responsible for chemoreception (gustatory receptors), were found. This study increases the available detailed morphological and behavioural data for Hemiptera and contribute to improve our understanding of these pests’ feeding behaviour and sensory mechanisms.

**Abstract:**

A detailed description of the labial sensory organs of *Leptoglossus occidentalis* Heidemann, 1910 and *L. zonatus* (Dallas, 1852) (Hemiptera: Heteroptera: Coreidae) is presented. The detailed morphology, location, and distribution of different sensilla types on mouthparts were investigated and shown in micrographs taken with a scanning electron microscope. Nine types of aporous sensilla, and three uniporous sensilla were found. The possible functions of these sensilla as well as similarities and differences between the mouthparts of *Leptoglossus* and those of other terrestrial Heteroptera are discussed. The tip of the labium constitutes a functional “touch and taste area”, combining the chemosensitivity of uniporous sensilla P1–P3 and the mechanoreceptivity of A8 and A9 hair-like sensilla. A set of two cone-like chemosensilla types (9 + 2) was found on each lateral lobe of the labial tip. Literature analysis showed that such a set of cone-like sensory organs on the labial tip may be common in terrestrial Heteroptera. This observation confirms that the number and arrangement of sensilla is conservative and can be important in diagnosing taxa at various levels and in phylogenetic studies based on morphology.

## 1. Introduction

Heteroptera (or true bugs) is a monophyletic suborder within Hemiptera. It has about 45,000 species with a habitat range unmatched by other groups, including aquatic and semiaquatic habitats [1,2]. Many heteropterans are of economic importance, such as some representatives of the genus *Leptoglossus* Guérin-Méneville, 1831 (Pentatomomorpha: Coreidae) [3], for instance.

*Leptoglossus* is a large genus with 62 species characterised by flattened and dilated hind tibiae [3] (hence the common name “leaf-footed bugs” of the family Coreidae), and the ability to form aggregations with the help of pheromones [4]. They are all phytophagous with a wide range of specialisations, from feeding on a single genus to extreme polyphagy [3]. The genus *Leptoglossus* is neotropical in origin, with most species limited to Central and South America [5,6]. Some species (including both in this study) cause substantial damage to crops and orchards [4].

**Figure 1:**
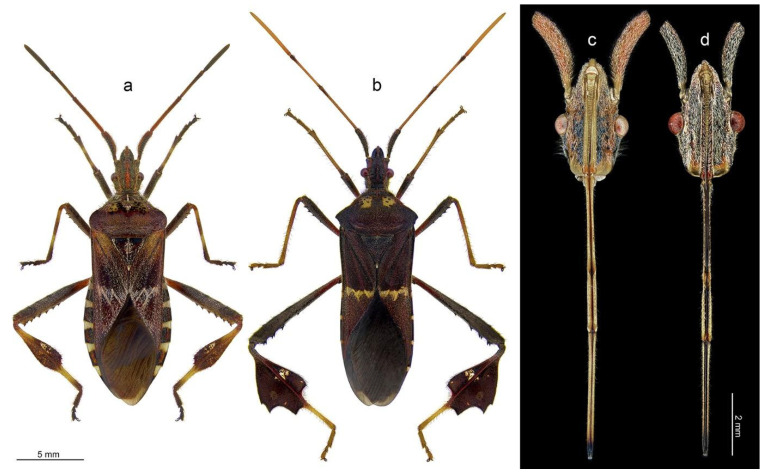
Dorsal habitus of examined species (**a**,**b**) and morphology of head with labium (**c**,**d**); *Leptoglossus occidentalis* (**a**,**c**), *Leptoglossus zonatus* (**b**,**d**).

*Leptoglossus occidentalis* Heidemann, 1910 [7] (Figure 1a), also called the western conifer seed bug, a native of western North America, has become a highly successful worldwide invader. Over the last six decades, this species has significantly expanded its range to include eastern North America, Europe, Asia Minor, eastern Asia, northern Africa, and South America [8,9]. This species feeds on conifer cones and can be a major pest in conifer seed orchards [4,10,11]. In Europe, it is of particular concern to growers of edible pine nuts, *Pinus pinea* L. [12].

*Leptoglossus zonatus* (Dallas, 1852) [13] (Figure 1b), or the large-legged bug, is a polyphagous species widely distributed throughout the Americas. *Leptoglossus zonatus* is an important pest of a wide range of crops in the southern United States, including cotton, tomato, eggplant, almonds, pistachios, pomegranate, peach, citrus, watermelon, corn, and pecan [3,4,14,15].

The sucking mouthparts in Hemiptera are often called the “rostrum”, “sucking beak”, or piercing-sucking mouthparts [16]. Throughout the evolutionary history of Hemiptera, the components of this feeding apparatus have been distinctly modified in different taxa, in order to serve unique functions in bugs adapted to feed from various food sources [17,18,19]. Compared to other groups of hemipterans, Heteroptera representatives display a particularly broad array of trophic and morphological diversity [18]. Morphological variation of insects’ mouthparts generally corresponds to their different feeding requirements [20,21]. Numerous sensilla of various types are attached to the different mouthparts, which play essential roles in host search, object detection, feeding, and mating [20,21,22]. The types and quantities of various sensilla are closely related to the feeding habits of insects [21]. Moreover, this diversity of sensilla in many insects is potentially a valuable information source for reconstructing their phylogeny [23,24].

Thus far, the morphology and ultrastructure of the labial receptors of true bugs have been studied in several species within families: Nepomorpha [18,25,26] Gerromorpha [18,27], Cimicomorpha [24,28,29,30,31,32,33,34,35,36,37], and Pentatomomorpha [32,38,39,40,41,42,43,44,45,46,47,48,49,50,51,52,53]. Within the Coreoidea superfamily, labial sensilla have been studied on a single species of Rhopalidae, *Rhopalus maculatus* (Fieber, 1837) [32], and two species of Alydidae: *Neomegalotomus parvus* (Westwood, 1842) [48] and *Riptortus pedestris* (Fabricius, 1775) [42]. In the case of Coreidae, data is very limited and refers to only one species, *Coreus marginatus* (Linnaeus, 1758) [32].

Thus, consistent and detailed studies are needed in order to provide valuable comparative data. This study aims to provide the first detailed morphological characterisation of the labial sensory organs of two *Leptoglossus* species using a scanning electron microscope (SEM). The morphology, location, and distribution of different sensilla types on mouthparts are investigated, and the possible functions of these sensilla are discussed.

## 2. Methods

### 2.1. Materials Examined

The study is based on two males, and two females of *L. occidentalis* collected in Poland (Upper Silesia) as well as a male and three females of *L. zonatus* from the neighbourhood of the town of Parlier, California (Fresno County). Specimens were picked up with tweezers and poisoned with ethyl acetate. The examined SEM preparations are preserved in the Institute of Biology, Biotechnology and Environmental Protection collections, Faculty of Natural Sciences, at the University of Silesia in Katowice (DZUS).

Because of the fact that no sexual dimorphism of the labial structure was found, it was decided to present the results without distinction of the sex of specimens.

### 2.2. Light Microscopy Procedures

The photographs (Figure 1) were taken following the method described by Taszakowski and Kaszyca [54]. To prepare high-quality photos that would enable advanced processing (e.g., obtaining a uniform background), the specimens were stuck onto transparent entomological glue boards and then cleaned with a thin brush. To improve the visibility of details, pictures of the labia were taken with the use of a dark background. The focus-stacked, colour photographs were captured using the following equipment: Leica M205C (stereomicroscope), Leica LED5000 HDI (high diffuse dome illumination), Leica DFC495 (digital camera), and Leica application suite 4.12.0 (software) (Leica Microsystems, Vienna, Austria). The resulting photographs were composed and enhanced with Image Composite Editor (panoramic image stitcher) and Adobe Photoshop CS6 graphic editor.

### 2.3. SEM Procedures

**Figure 2:**
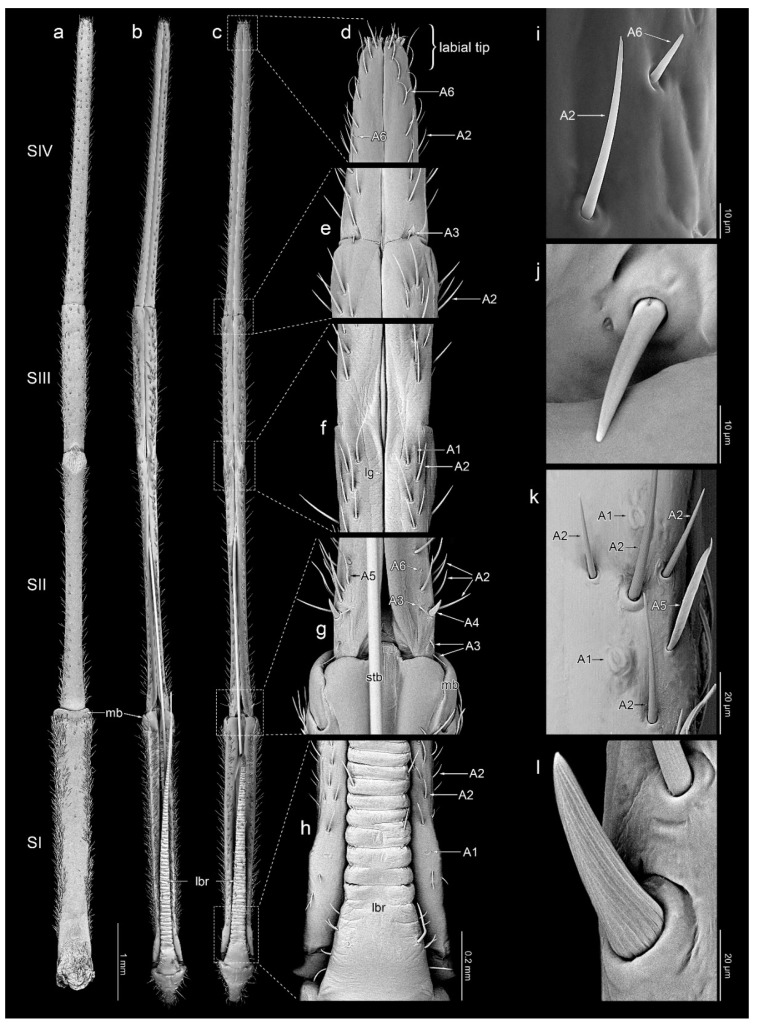
Detailed morphology of the labium; ventral view (**a**), dorsal view (**b**–**d**); detailed structure of selected parts of labium: apical part (**d**), connection of the segment III and IV (**e**), connection of the segment II and III (**f**), connection of the segment I and II (**g**), basal part of labium (**h**); particular sensilla in magnification: A2 and A6 (**i**), A3 (**j**), A1, A2, A5 (**k**), A4 (**l**). *Leptoglossus zonatus* (**a**,**b**,**k**), *Leptoglossus occidentalis* (**c**–**j**,**l**). Abbreviations: A1–A6—types of sensilla; lg—labial groove; lbr—labrum; mb—membrane; SI–SIV—numbers of labial segments; stb—stylet bundle.

**Figure 3:**
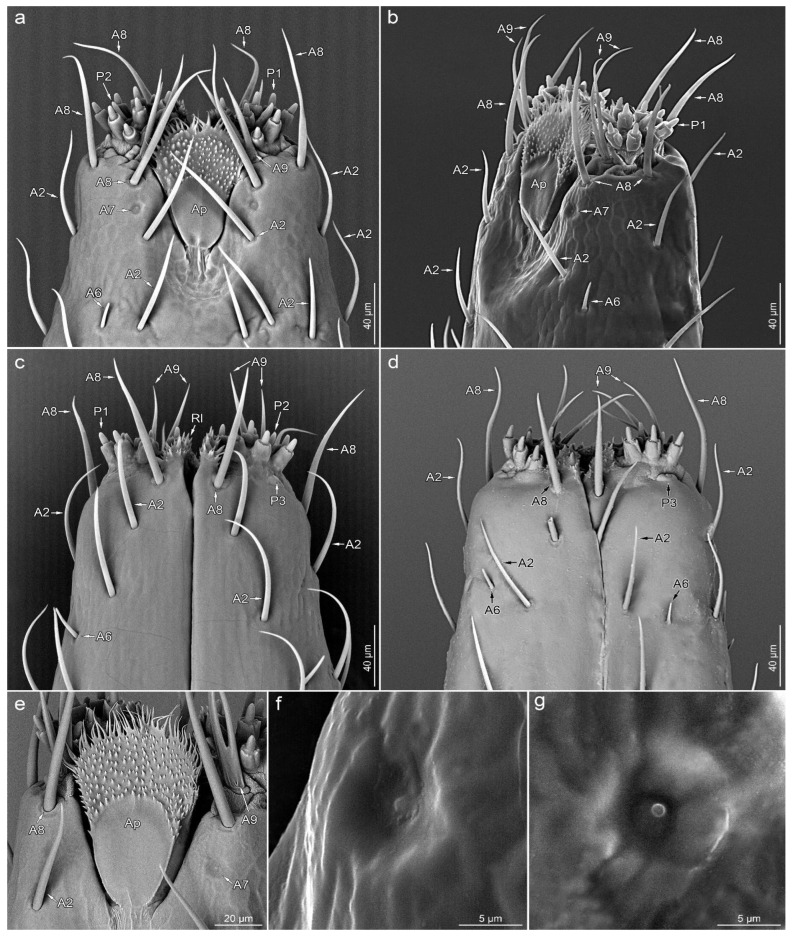
Detailed morphology of labial tip; ventral view (**a**), ventrolateral view (**b**), dorsal view (**c**,**d**), apical plate (**e**), sensillum type A7 in magnification (**f**,**g**). *Leptoglossus occidentalis* (**a**–**c**,**e**–**g**). *Leptoglossus zonatus* (**d**). Abbreviations: A2, A6–A9, P1–P3—types of sensilla; Ap—apical plate; Rl—rostral lid.

**Figure 4:**
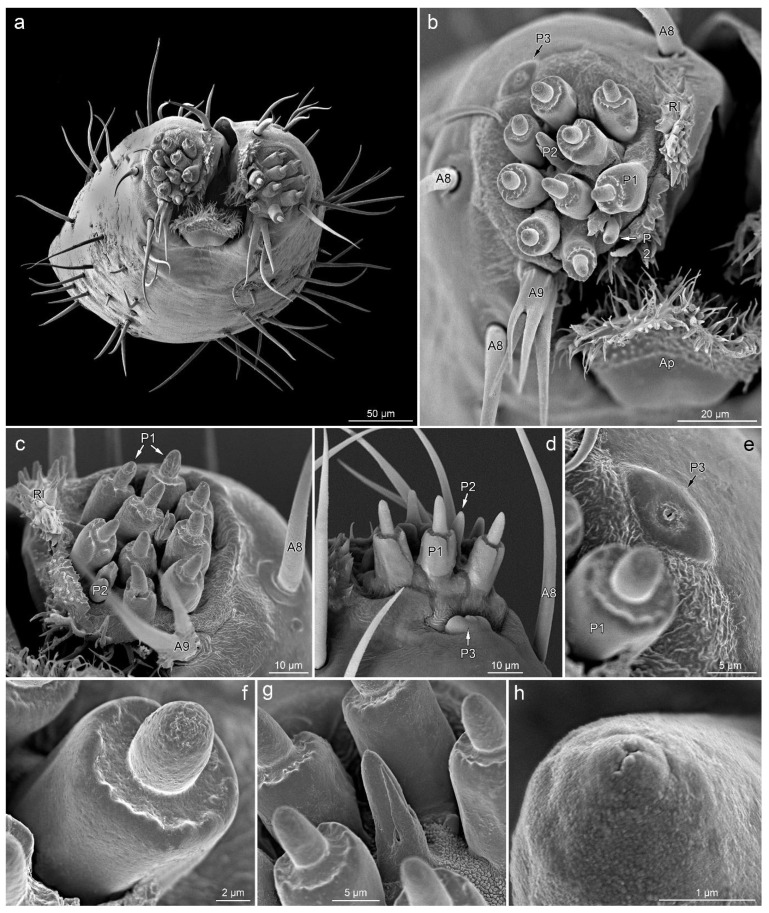
Distribution of sensilla on the labial tip; anterior view (**a**), right lateral lobe in anterior view (**b**), left lateral lobe in ventro-anterior (**c**) and dorsal view (**d**), details of sensillum P3 (**e**), details of sensillum P1 (**f**), sensillum P2 surrounded by sensilla P1 (**g**), pore on the apex of sensillum P2 (**h**). *Leptoglossus occidentalis* (**a**–**c**,**e**–**h**). *Leptoglossus zonatus* (**d**). Abbreviations: A8, A9, P1–P3—types of sensilla; Ap—apical plate; Rl—rostral lid.

The material was dissected to separate the labia from the head and cleaned in detergent using an ultrasonic cleaner. Then, the standard procedure was applied [54]: dehydration with a series of baths in 80%, 90%, and 96% ethanol solutions, for 20 min each, and two baths of 99.8% ethanol solution for 30 min each. The labia were glued with carbon adhesive discs on the aluminium pin stubs, which then were coated with a film of gold (30 nm) using the Q150T ES sputter coater with the rotary planetary stage (Quorum Technologies Ltd., Laughton, UK). SEM micrographs (Figure 2, Figure 3 and Figure 4) were obtained using a Phenom XL field emission scanning electron microscope (Phenom-World B.V., Eindhoven, The Netherlands) at 15 kV accelerating voltage and with a BackScatter Detector (BSD) and Secondary Electron Detector (SED) and Hitachi UHR FE-SEM SU8010 (High Technologies, Tokyo, Japan) with a secondary-electron detector (ESD) at 5, 7, and 10 kV accelerating voltage. To obtain high-quality figures, fragments of labia were imaged at high magnifications and combined using the Image Composite Editor (panoramic image stitcher) and the graphic editor Adobe Photoshop CS6. In a few cases, a series of images at different focal distances were taken and combined using the software mentioned above to attain the appropriate depth of field.

### 2.4. Measurements

The lengths of the labia and their particular segments were measured using an electronic vernier by Leica application suite 4.12.0 software and presented in millimetres (mm).

### 2.5. Terminology for the Sensilla

The identification of sensilla and the terminology used in the present study were based on the main classifications according to Altner and Prillinger [55] and Shields [56], as well as morphological comparisons of the antennal sensilla of different species of Heteroptera presented in several papers [24,49,51,52].

## 3. Results

Since the structure of the labium and the sensory organs of both species showed no differences, they are discussed together.

### 3.1. Morphology of Labium

The labium is elongated, slender (an average of 11.33 mm in *L. occidentalis* and 11.56 mm in *L. zonatus*), and four-segmented (Figure 1c,d and Figure 2a–c). No sexual dimorphism of the labial structure was found. The approximate lengths of particular segments are as follows: in *L. occidentalis* SI: 2.94 mm, SII: 2.93 mm, SIII: 1.81 mm, SIV: 3.65 mm; and in *L. zonatus* SI: 3.12 mm, SII: 3.11 mm, SIII: 1.82 mm, SIV: 3.51 mm (*n* = 4 in both species). Except for the partially fused dorsal surface SII and SIII (Figure 2f), the boundaries between the segments are clearly visible. All segments have a similar cylindrical shape. The apical part of segment IV is slightly tapered. Its end, referred to as the “labial tip”, is tripartite, distinctly divided into two lateral lobes with an apical plate on the ventral side (Figure 3a–e and Figure 4a). The dorsal surface of the labium is deeply concave, forming a groove that contains the mandibular and maxillary stylets (Figure 2a–c). On the basal part of the dorsal surface, the labial groove is wide and open enough to accommodate the labrum.

### 3.2. Sensilla Types

In accessible literature, the terminology regarding various types of sensilla is based on their shapes and sizes (length). The names of different morphological types of sensilla, particularly “trichoid”, “chaetic”, and “basiconic”, are used variably and interchangeably by various authors. In the current categorisation of sensilla, length is not an important criterion for classification because of the significant discrepancies in the descriptions of sensilla in the heteropteran species by different authors. Therefore, we have decided to simplify the nomenclature and include the sensilla’s features in their descriptions.

#### 3.2.1. Aporous Sensilla

**A1**—oval and cupola-shaped structure, not evidently protruding over the surface of the cuticle (Figure 2f,h,k).

**A2**—hair-like sensillum with a finely grooved wall, embedded in a flexible socket. These sensilla taper evenly along its entire length, protrude over the surface of the cuticle and are directed along the labium’s long axis. Type A2 includes sensilla that differ slightly in shape, size, and orientation (Figure 2d–i,k and Figure 3a–e).

**A3**—smooth, cone-shaped sensillum arising perpendicularly from the surface of a flexible socket. The stem of the sensillum is stiff and blunt-ended (Figure 2e,g,j).

**A4**—embedded in a flexible socket, robust, cone-shaped sensillum with a distinctly grooved wall (Figure 2g,l).

**A5**—smooth, flattened, hair-like, and fine-ended sensillum embedded in a flexible socket. The base of the sensillum is narrower than the distal part (Figure 2g,k).

**A6**—smooth, short cone-like sensillum. The sensillum stem is stiff and sharp at the end. These sensilla are protruding and oriented at a small angle to the surface of the labium (Figure 2d,g,i and Figure 3a–d).

**A7**—shallow depression in the cuticle with a centrally located short process (Figure 3a,b,e–g).

**A8**—embedded in a flexible socket, hair-like sensillum with a very finely grooved wall. The basal 2/3 of the length of the stem is evenly thick, with the distal part tapering towards the apex (Figure 3a–e).

**A9**—double or quadruple hair-like sensillum, with a shared basal part embedded in a single flexible socket. Smooth stems are slightly flattened at the base and sharp at the ends (Figure 3a–e).

#### 3.2.2. Porous Sensilla

**P1**—short cones with a single pore on a rounded apex. These sensilla are embedded in inflexible sockets, which are located on unusual, elongated bases (Figure 3a–c and Figure 4b–g).

**P2**—short cones tapered slightly from the base to the blunt apex with a terminal pore, partially enclosed by fused cuticular processes, embedded in an inflexible socket (Figure 3a,c and Figure 4b–d,g,h).

**P3**—oval and cupola-shaped structure, slightly protruding over the surface, with a single central pore (Figure 4b,d,e).

### 3.3. Sensilla Arrangement

#### 3.3.1. Segment I

Dorsal surface: there is a pair of symmetrically located (on each side of the groove) sensilla A1 and a few scattered sensilla A2 on the basal part. Excluding the distal end, the rest of the segment is covered with sensilla A2 and A5. Ventral surface: almost glabrous; only the side edges are covered with sensilla A2 and A5.

#### 3.3.2. Segment II

Dorsal surface: on the basal part of the segment, there are three symmetrically located pairs of sensilla A3 and a pair of sensilla A4. Additionally, in the basal part between the sensilla A2, there are scattered sensilla A5. Along the entire length of the segment, there is a single row of sensilla A2 on both sides of the labial groove. Between them, there are a few asymmetrically distributed sensilla A6. At the distal end of the segment, there are two pairs of symmetrically arranged sensilla A1. Ventral surface: almost glabrous along the midline; the sides are covered with sensilla A2 and A5.

#### 3.3.3. Segment III

Dorsal surface: covered with numerous sensilla A2 and A5, and a few sensilla A6. Ventral surface: except for the midline, covered sparsely with short sensilla A2. The sides are covered with sensilla A2 and A5.

#### 3.3.4. Segment IV

Dorsal surface: at the segment’s basal edge, there is a symmetrically located single pair of sensilla A3. Along the entire length of the segment, there is a single row of sensilla A2 on both sides of the labial groove. Between them, in the distal half, there are a few sensilla A6. Ventral surface: covered sparsely with short sensilla A2 along its entire length. A few A6 sensilla are also present in the distal part.

Labial tip

The lateral lobes of the labial tip consist of two symmetrical sensory fields. Below the lateral lobes, on the sides of the base of the apical plate, there is a pair of symmetrically located sensilla A7. Around the base of each lateral lobe, there are three sensilla A8 (one on each ventral, lateral and dorsal sides), a single sensillum A9 (on the ventral side, outside the distal part of the apical plate) and a single sensillum P3 (near the outer edge on the ventral side). At the top of the lateral lobe, there are nine sensilla P1, with two sensilla P2 in between.

### 3.4. Other Cuticular Structures

**Apical plate (Ap)**—trapezoidal plate (Figure 1a,b,e and Figure 4a,b), which is contained within the ventral groove and forms a rostral lid, apparently lacking sensory organs. The basal part of the apical plate is smooth; the distal part is covered with short, cuticular microtrichia. The distal edge and internal surface are covered with long cuticular projections.

**Rostral lid** (**Rl)**—is a pair of cuticular protuberances covered by numerous cuticular processes of various shapes (Figure 4a–c). They are located at the inner side of each sensory lobe, delimiting the space between them and the labial groove.

## 4. Discussion

### 4.1. Mechanoreception

Insects can receive and respond to numerous different types of mechanical stimuli. These include touch, air currents, sound, gravity, and deformations of body regions that may be caused either by external forces or by self-produced movements [57]. Two main groups of mechanoreceptors [57] were observed on the labium of the studied insects: the campaniform sensilla (A1) and the bristles (A2–A6, A8, A9) (Table 1). The first mentioned sensilla detect the deformation of the cuticle, whereas the bristles perform tactile functions. Sensilla A3 act as proprioceptors, as indicated, among others, by their morphology, location, and orientation. The other types of cone- and hair-shaped sensilla (A2, A4 –A6, A8, A9) are probably responsible for the detection of external objects. The robust sensilla A4 are noteworthy. It seems that they can additionally perform a typical mechanical (structural) function. Also observed in this study, sensilla A7 have a peculiar structure which has not been described in other studies. They are somewhat similar to campaniform sensilla, but further research is necessary to determine their function and whether they actually are sensory organs.

*Leptoglossus*’ mechanosensilla (Table 1) do not differ in structure and distribution from related species [49,51]. Compared to other taxa, e.g., Pentatomidae [47,52] and Miridae [35], mechanoreceptors of *Leptoglossus* show little morphological diversity. Unfortunately, the analysis of sensilla along the entire length of the labium has been carried out in only a few species, which makes it difficult to perform a large-scale comparative analysis.

It should be noted that further studies, including electron transmission microscopy observation or electrophysiological and behavioural bioassays, are necessary to confirm the mechanoreceptive as well as the chemoreceptive function assigned here (see below).

### 4.2. Chemoreception

Chemoreceptors are responsible for insects’ senses of taste and smell. The olfactory receptors detect gaseous chemicals, and the gustatory receptors detect compounds dissolved in water or even on dry surfaces [20,55,58]. Sensilla responsible for the perception of taste sensations usually have one pore in their apical part [20,55,58,59]. Olfactory sensilla are usually densely covered with numerous pores over almost the entire surface [20,55,58,59]. The three types of chemoreceptors (P1–P3) found on the labial tip of the studied specimens have a single pore at the apex, indicating that they act as gustatory receptors.

Sensilla P1 occur in many terrestrial Heteroptera and are most often referred to as sensilla basiconica [24,28,29,32,33,35,36,38,40,42,43,44,45,46,47,49,51,60] or sensilla [51,52]. Compared to other species, this type of sensilla in *Leptoglossus* is characterised by a unique, long base (called raised platform or cylindrical socket). Not all authors distinguish between P1 and P2 sensilla. However, high-quality micrographs in papers on species from various taxonomic groups allow for the analysis of sensilla. Within the Cimicomorpha, these are: Miroidea, Miridae: *Lygus lineolaris* [28,29], *L. rugulipennis* [33], *Adelphocoris fasciaticollis* [60], and *Cheilocapsus nigrescens* [35]; Miroidea, Tingidae: *Stephanitis nashi* [36]; Naboidea, Nabidae: *Himacerus apterus*, *H. mirmicoides,* and *Prostemma guttula* [24]. Within the Pentatomomorpha: Pyrrhocoroidea, Pyrrhocoridae: *Pyrrhocoris sibiricus* [49]; Pyrrhocoroidea, Largidae: *Physopelta cincticollis* [51]; and Pentatomoidea, Pentatomidae: *Cazira bhoutanica, Picromerus bidens,* and *P. lewisi* [52]. In all the above cases, as with *Leptoglossus*, there are nine sensilla P1 on each lateral lobe. It is widely recognised that sensilla P1 are contact chemoreceptors and therefore have a gustatory function. This is evidenced by the presence of terminal pores and ultrastructural details [33].

Sensilla P2 are also present in all of the above species (always two), although, as already mentioned, they are not always distinguished. Similar to the P1 type, sensilla P2 are most often considered to be uniporous contact chemoreceptors. However, in the case of *Eocanthecona furcellata* (Pentatomidae), sensilla basiconica type B’s (equivalent to type P2) surface is perforated with numerous pores, which indicates olfactory function [46]. Furthermore, in *Dysdercus fasciatus* (Pyrrhocoridae), Peregrine [38] suggested the presence of numerous pores on the surface of basiconic sensilla (however, he did not distinguish two subtypes of sensilla). On the other hand, Taszakowski et al. [24] assigned the nonporous peg sensilla (= aporous styloconic sensillum) of nabids as thermo-hygroreceptors. However, it is possible that more detailed studies of these sensilla would reveal apical pores, which would indicate a gustatory function. In several studied damsel bugs (Nabidae) species, these sensilla have an unusual morphology [24]. Either they did not stick out much, or they were hidden inside the base. It seems possible that the base surrounding the sensillum results from the fusion of cuticular processes that can be observed in *Leptoglossus* and *P. sibiricus* [49] or *Dolycoris indicus* [47].

Sensilla P3 does not seem to have an equivalent in Heteroptera’s representatives studied so far. They look a bit like campaniform sensilla but stand out clearly above the surface and have a large, centrally located pore. In addition to the previously mentioned P1 and P2 sensilla equivalents, various sensory organs were observed on the labium tip of heteropterans. These include, e.g., oval plate sensilla with a terminal pore and placoid elongated sensilla with wall pores, which have been shown in Nabidae [24], multiporous sensilla placodea, and “flower-like sensilla” in lace bug *S. nashi* [36]. The mentioned sensilla appear singularly on each lateral lobe. Moreover, in *P. sibiricus*, a single sensillum basiconicum IV on each lateral lobe [49] (no. 4 in Figure 7E) appears to represent a different type.

It has already been noticed before that true bugs show little variation among species in terms of the number and arrangement of sensilla on the labium [33,41]. On the other hand, detailed research reveals an increasing number of minor differences which may, however, be of crucial functional importance. Representatives of the genus *Leptoglossus* are relatively large insects, and the details of the tip of the labium are easy to observe. However, for many small bugs, especially those with delicate, sharpened labia, conducting of detailed morphological research is a real challenge. Furthermore, in some large bugs, dense hair-like sensilla obscure the chemoreceptors on the labial tip [50].

In turn, in Reduviidae, a highly specialised labium is crucial for stridulation, which is obtained by rubbing the tip of the labium against the transversely striate prosternal sulcus. There are two small, heavily sclerotised tubercles called plectrum on each lateral lobe [61]. For this reason, the sensilla on the pointed tip of the labium are short or flat [30,34], so they do not interfere with stridulation. In such cases, establishing the homology is difficult. The characteristic set of chemosensilla: 2 × (9 + 2) (two lateral lobes, nine P1 and two P2 sensilla on each lobe) on the tip of the labium in the analysed species confirms that the number and arrangement of sensilla are conservative. Therefore, they can be important in diagnosing taxa at various levels and in phylogenetic studies based on morphology. Literature analysis showed that such a cone-like sensory organs on the labial tip may be common in terrestrial Heteroptera.

## 5. Conclusions

The elongated and slender, four-segmented labia have the same structure in both analysed species. Nine types of aporous sensilla whose probable function is mechanoreception and three uniporous sensilla most likely responsible for chemoreception (gustatory receptors) were found. The tip of the labium constitutes a functional “touch and taste area”, combining the chemosensitivity of uniporous sensilla P1–P3 and the mechanoreceptivity of A8 and A9 hair-like sensilla. The set of two cone-like chemosensilla types (9 + 2) on each lateral lobe of the labial tip was found. Literature analysis showed that such cone-like sensory organs on the labial tip may be common in terrestrial Heteroptera. This observation confirms that the number and arrangement of sensilla are conservative and can be important in diagnosing taxa at various levels and in phylogenetic studies based on morphology. It should be noted that further studies, including electron transmission microscopy observation or electrophysiological and behavioural bioassays, are necessary to confirm the functions of the sensory organs proposed here.

## Figures and Tables

**Table 1:** Characteristics of the identified types of sensory organs. SI–SIV—numbers of labial segments (lt)—labium tip.

Aporous Sensilla				
Type	Localisation	Shape	Cuticle Surface	Proposed Function
A1	SI, SIII	cupola	–	mechanoreception
A2	SI, SII, SIV	hair-like	grooved	mechanoreception
A3	SII, SIV	cone-like	smooth	mechanoreception (proprioreception)
A4	SII	cone-like	grooved	mechanoreception
A5	SI, SII, SIII	hair-like	smooth	mechanoreception
A6	SII, SIII, SIV	cone-like	smooth	mechanoreception
A7	SIV (lt)	shallow depression	–	mechanoreception
A8	SIV (lt)	hair-like	finely grooved	mechanoreception
A9	SIV (lt)	hair-like	smooth	mechanoreception
**Porous Sensilla**				
**Type**	**Localisation**	**Shape**	**Cuticle**	**Proposed Function**
P1	SIV (lt)	cone-like	smooth	chemoreception (gustation)
P2	SIV (lt)	cone-like	smooth	chemoreception (gustation)
P3	SIV (lt)	cupola	–	chemoreception

## Data Availability

All data generated or analysed during this study are included in this published article.
